# Wendigo Psychosis and Psychiatric Perspectives of Cannibalism: A Complex Interplay of Culture, Psychology, and History

**DOI:** 10.7759/cureus.47962

**Published:** 2023-10-30

**Authors:** Sean E Oldak, Anthony J Maristany, Brianna C Sa

**Affiliations:** 1 Psychiatry and Behavioral Sciences, University of Miami Miller School of Medicine/Jackson Memorial Hospital, Miami, USA; 2 Psychiatry and Behavioral Sciences, University of Miami Miller School of Medicine, Miami, USA

**Keywords:** indigenous tribe, native american, culture-bound syndrome, psychosis, wendigo

## Abstract

Our review paper delves into the intricate and multifaceted realm of cannibalism, with a focused exploration of its manifestations in Wendigo psychosis. We aim to explore the implications of cannibalism within the realms of psychiatry, anthropology, psychology, and sociology by navigating the complexities of cultural beliefs, psychological underpinnings, historical contexts, and contemporary significance surrounding cannibalism.

Cannibalism is deeply ingrained in the cultural and mythological heritage of Algonquian-speaking tribes; it is closely associated with the symbolic figure of the Wendigo. The Wendigo serves as a warning about the potential loss of one's humanity in dire circumstances like starvation. Wendigo psychosis, characterized by psychiatric manifestations such as paranoia, anxiety, hallucinations, and cannibalistic urges, often emerges as a result of a fusion of cultural narratives and psychological vulnerabilities. This may provide an outlet for individuals experiencing internal distress. Historical records show that instances of Wendigo psychosis and cannibalism were more prevalent during periods of extreme scarcity and famine among Algonquian tribes, but they can also manifest in non-famine contexts.

Cannibalism assumes diverse forms and meanings across various cultures, encompassing ritualistic, sacrificial, or survival cannibalism. Acknowledging these nuances is paramount to avoiding perpetuating harmful stereotypes and to appreciating the significance of these practices within specific cultures. Engaging in discussions about cannibalism necessitates cultural sensitivity and respect for diverse cultural practices and beliefs to foster open dialogue and enhance cross-cultural understanding.

Although cannibalism is often associated with psychiatric disorders, it is not exclusively rooted in mental illness. Factors like substance abuse, antisocial traits, and environmental upbringing can also contribute to cannibalistic acts. In some cases, cannibalism may be linked to survival instincts stemming from trauma and abuse. Therefore, it is vital to distinguish between various forms of cannibalism and understand their underlying motivations. Analyzing cannibalistic fantasies from a psychoanalytic perspective involves exploring mechanisms such as melancholia and oral fixation, shedding light on the psychological underpinnings of these thoughts and urges.

Moreover, the influence of media portrayals of cannibalism on public perceptions cannot be underestimated. Sensationalism and romanticization in popular culture can distort our understanding of the motivations and mental states of individuals involved in cannibalistic acts.

In essence, cannibalism remains an intriguing and multidimensional topic deeply entrenched in cultural narratives and psychological complexities. A comprehensive understanding necessitates a multidisciplinary approach, taking into account how historical context, cultural beliefs, psychological experiences, and societal dimensions shape human behavior and our comprehension of the human condition. To navigate this complex subject with sensitivity and respect, it is essential to recognize the diverse manifestations and motivations behind cannibalistic behavior, whether in the context of Wendigo psychosis or other cultural practices.

## Introduction and background

Cannibalism garners significant intrigue as its manifestation is multifaceted and explores the boundaries of human behavior, ethics, and morality. The act of consuming human flesh has been documented throughout history and across various cultures, raising profound questions about the underlying psychopathology and motivations of those who commit such acts. It is important to note that the practice of cannibalism is not a homogenous behavior; rather, it encompasses a range of practices, each with its own psychological basis. One of the major and fundamental questions in the psychology of cannibalism is the motivation behind such an act. If we view the act of cannibalism in regard to motivation, there are three major categories that may be elucidated: nutritional, ritual, and pathological [1]. Nutritional cannibalism is linked to the use of human flesh strictly for its caloric content and has been seen more readily in times of acute starvation, such as in times of extreme situations such as famine or when stranded in life-threatening conditions, in which survival is paramount [1]. Ritual cannibalism is linked to cultural practices that require or contain cannibalism as part of the practice, such as part of religious or funerary rites. In the case of ritual cannibalism, the act of consuming human flesh is intertwined with cultural beliefs and norms, challenging the idea of what is considered culturally acceptable by certain cultures [1]. Pathological cannibalism is rooted in some form of psychopathology that is at the core of the person’s motivation, such as someone who is acutely psychotic or committing the act of cannibalism to act out paraphilia [1]. It is in the realm of pathological cannibalism that most criminal acts of cannibalism are discussed. Given the psychopathological component of these acts, the field of psychiatry has investigated this topic from a clinical and forensic point of view, and this has expanded to include discussion about treatment.

As detailed above, cannibalism is a phenomenon replete with a diverse array of psychopathological underpinnings. While some cases can be specifically attributed to underlying psychiatric conditions, others are influenced by a complex interplay of environmental factors, sociocultural dynamics, and individual psychodynamics. In this paper, we explore the intricate subject of cannibalism through a lens that encompasses cultural sensitivity, psychoanalytic perspectives, and its representation within popular media. We aim to approach this often taboo subject with sensitivity and nuance to shed light on the complex motivations and behaviors associated with cannibalism while transcending the limitations of sensationalism and stereotypes. In the process, we engage in a thoughtful exploration of the multifaceted interplay between myth, culture, psychology, and human behavior. To this end, we will explore a rare but fascinating phenomenon known as Wendigo psychosis, in which cannibalism is a core finding.

Wendigo (also known as windigo, whitiko, wihtigo, wihtiko, witigo, witiko, or wittingo) psychosis is a fascinating and enigmatic culture-bound syndrome rooted in the mythologies of Algonquian-speaking tribes, particularly those in the northern regions of North America [2]. This phenomenon is marked by an overwhelming belief and delusion that individuals are transforming into Wendigos, malevolent spirits driven by an insatiable desire for human flesh [2]. This myth reflects the famines and hardships endured by the Algonquian community, making it a powerful narrative that continues to influence psychological experiences within these communities [2]. Wendigo psychosis, marked by symptoms such as paranoia, anxiety, and cannibalistic urges, provides a unique opportunity to delve into the psychological underpinnings of cultural beliefs and coping mechanisms as well as their relation to cannibalism [2-4]. In this paper, we will delve into the multilayered aspects of Wendigo psychosis and its connection to cannibalism, examining it in the context of cultural beliefs, psychological underpinnings, historical manifestations, and contemporary implications.

## Review

Cultural origins and mythological roots

Algonquian-speaking tribes, including Cree, Ojibwa, and other Algonquian-speaking tribes of North America, possess rich mythologies marked by a complex web of belief systems, symbolism, and narratives that shape their collective understanding of the world. The Wendigo holds a significant place in Algonquian mythologies, symbolizing a malevolent spirit characterized by insatiable hunger and cannibalistic tendencies [2]. This being manifests as a towering, emaciated figure emanating the odor of decaying flesh and an aura of terror [2]. Additionally, the Wendigo has been described as having fiery eyes, a heart made of ice, and the ability to transform other humans into Wendigos [3-5]. The Wendigo encapsulates and represents the dread of limited resources during harsh winters and the dangers of yielding to personal greed. Its gaunt form and ice heart reflect the famines and hardships endured by the Algonquian community in severe winters [4]. The Wendigo's insatiable appetite mirrors the desperation that emerges amidst scarcity, while its transformation from human to monstrous entity signifies the risk of losing one's humanity in dire situations [5]. Functioning as a cautionary story, the Wendigo serves as a reminder of the pitfalls of forsaking communal values in favor of self-centered desires. These mythologies have a profound impact on psychological experiences in Algonquian communities, shaping how individuals understand and respond to their psychological distress across generations. Wendigo psychosis may arise from the fusion of cultural narratives and psychological vulnerabilities, where the belief in transformation into a Wendigo serves as an outlet for expressing internal distress [3,6].

Cultural beliefs, psychological underpinnings, and coping mechanisms

Individuals experiencing Wendigo psychosis exhibit a range of symptoms, including paranoia, anxiety, hallucinations, and cannibalistic urges. Typically, the onset of Wendigo psychosis is marked by depression, nausea, and anorexia [7]. As the disorder advances, individuals begin to believe that they are possessed by the Wendigo, leading to heightened levels of paranoia and violent hallucinations of potential victims in the form of animals to be killed and devoured [7]. Further along, those afflicted may develop an alarming shift in perception, viewing others, even close family members, as potential prey [7]. Once human flesh is consumed, the transformation to Wendigo is considered complete and irreversible [7].

Psychological theories that appreciate coping mechanisms, externalization, and their interplay with cultural context are crucial to understanding the manifestation of Wendigo psychosis. Coping mechanisms are cognitive and behavioral strategies employed to navigate both internal and external struggles and are influenced by the cultural milieu, including how individuals perceive stressors, express distress, and seek support. Paredes claims that societal narratives, especially those crafted in mythology or literature, create a “cognitive map” from which our subconscious can trace an individualized fantasy and seek refuge [8]. These frameworks assist individuals in navigating their experiences, often shaping narratives that both reflex and externalize their context and conflicts. This theory is exemplified in the case described by Paredes of a woman from northern Minnesota with a history of profound hardship but also exposure to Ojibwa culture, in which she describes her lifelong dreams of the Wendigo [8]. In her dreams, the Wendigo took on different figures and roles: in her youth, serving as a protector, and in her later life, signifying self-identity and resilience [8]. The shifting roles of the Wendigo in her dreams appear to mirror her journey through challenges and self-discovery. These dreams draw upon the mythological attributes of the Wendigo in a way that allows her to process her experiences, encapsulating the dual impact of adversity and cultural heritage on her psyche [8].

Similarly, in the Ojibwa culture, coping mechanisms are intricately linked to the early socialization and rearing processes [7]. In the developmental phase of childhood, Ojibwa children undergo a transition from nurturing care to instruction focused on attaining power, love, and commendation through acts of sacrifice [2,7]. From a young age, affection and acceptance become contingent on fasting and isolation rituals [2,7]. A prevalent practice involves presenting a child with a dilemma: the option to choose between food and charcoal. Opting for food results in punitive measures, whereas selecting charcoal leads to rewards [7]. The drastic shift from nurturing rearing to a self-sustaining and sacrificing practice can create dependency cravings [2,7]. Additionally, the early association of pain with pleasure may lay the groundwork for masochist tendencies to develop [7]. In older Ojibwa individuals, the concept of restraint is intertwined with the power and status obtained from procuring food [7]. The societal structure also emphasizes individual achievements over communal advancement, fostering competition and individualism [7]. Cannibalism could potentially function as a maladaptive coping mechanism for individuals navigating psychological trauma within the above context. It might emerge as a strategy to navigate the delicate interplay between sustenance and control, emotions of inadequacy and acceptance, and security and independence. Moreover, it could serve as an attempt to restore a sense of power or attention that is perceived as having been stolen, forfeited, or never achieved through extreme actions. Interestingly, the use of self-cannibalism to gain power has also been documented in the case of a 29-year-old homeless man [9]. This individual was admitted to a hospital after trying to consume his own finger, a gesture aimed at demonstrating his psychological dominance and distinctive nature and to discourage potential harm from others [9].

Additionally, the development of Wendigo psychosis can further be understood through the concept of externalization. Externalization is a psychological defense mechanism that is not just restricted to this specific cultural phenomenon but also a universal feature of human experience [10]. Through externalization, individuals can project their feelings onto external factors, situations, or individuals to cope with confronting their inner turmoil and negative emotions directly [10]. By attributing cannibalistic desires to Wendigo's transformation, individuals distance themselves from the discomfort of confronting their conflicting emotions and the social stigmatization associated with cannibalism.

The extreme conditions of starvation and survival push individuals to their physical and emotional limits, making it almost unbearable to acknowledge their own cannibalistic thoughts. By attributing these thoughts to the mythical creature, the Wendigo, individuals create a psychological buffer that shields them from the full weight of their actions. This not only offers a means of emotional survival but also provides a cultural context for their behavior, reducing stigmatization within their community.

This phenomenon highlights the complex interactions between culture, psychology, and survival instincts by demonstrating how individuals can adapt to extreme circumstances through the lens of folklore and mythological underpinnings. This reinforces the idea that human behavior is often shaped by an amalgamation of internal and external stressors, with externalization being just one of many mechanisms we may employ to navigate the challenges of life.

Historical and cultural context

In contemporary times, Wendigo psychosis has become a rarity, assuming a prominent role as a historical, cultural, and mythological construct due to the increased availability of psychiatric interventions and more stable food supplies [2]. The emergence of Wendigo psychosis among Algonquian tribes spans from the 18th century to the 19th century and appears to be rooted in intricate interactions between cultural mythologies, environmental exigencies, and human experience [5]. The Algonquian tribes, originating from Canada, represent indigenous communities practicing a hunter-gatherer lifestyle heavily dependent on the land for sustenance [7]. However, this dynamic becomes particularly challenging amid the prolonged, severe, and unforgiving winters, which are characterized by limited resources [7]. These conditions create a notable scarcity, increasing the community's vulnerability to experiencing episodes of famine [2]. Cannibalism, and indirectly, Wendigo psychosis, can become a maladaptive and individualistic survival strategy during times of extreme scarcity. Although rare, historical records highlight time periods where periods of famine aligned with the development of Wendigo psychosis and cannibalism dating back to 1741 [5]. In 1786, an Obijawa man killed and consumed his relatives during a famine, and in 1879, an elderly Cree woman developed mania and cannibalistic tendencies during a famine [5]. Encouragingly, the elderly woman’s condition showed marked improvement following enhanced care and improved nutritional intake [5]. Providing adequate nutritional support to someone going through a famine state and experiencing Wendigo psychosis is not only a humane and compassionate course of action but also a potentially effective means of improving psychotic pathology. This approach is rooted in the notion that malnutrition can illicit aggression and agitation in individuals, and, therefore, providing nutritional support can reduce these negative behaviors, make it easier to manage the individual's condition, and create a safer environment for both them and their caregivers [5]. Additionally, a person experiencing Wendigo psychosis may not be receptive to treatment when in a state of extreme hunger and distress. Addressing their immediate nutritional needs creates a foundation for them to engage more effectively in therapeutic interventions, which are crucial for improving their psychotic pathology [5]. While famine can serve as a catalyst for the development of Wendigo psychosis, it is not a universal driver, as there are cases of non-famine Wendigo psychosis [5].

Cannibalism as a cultural taboo

In addition to ritualistic presentations, cannibalism can also manifest in other non-criminal modalities, such as sacrificial or survival cannibalism [11]. While these three forms of cannibalistic practices are often considered cultural taboos within Western ideologies, they were historically embraced in various cultures as customary traditions or survival strategies.

Although cannibalism is rare, primarily due to its association with the transmission of pathogens in diseases such as kuru, certain genetic polymorphisms within human populations have been proposed as evolutionary protective mechanisms, hinting at a more common prevalence of cannibalism in earlier stages of human evolution [1]. According to Mead et al., those survivors of the Kuru epidemic who were predominantly heterozygous for the human prion protein gene (PRNP) 129 were relatively resistant to PRNP 129 homozygotes. It is possible that those who were heterozygous for this polymorphism were less likely to acquire Kuru or other pathogens of disease if they engaged in endocannibalistic feasts [12].

Furthermore, anthropological studies have explored historical instances of cannibalism, often involving a response to specific social and survival circumstances or as part of rituals [1,13-14]. Notable examples include documented cases of cannibalism in the twentieth century from tribes in Papua New Guinea, South America, and Africa [15]. Historical records also highlight instances of cannibalism during severe famine in both World Wars [1]. A more recent example is the crash of the Uruguayan Air Force flight 571 in 1972, where stranded passengers survived by feeding on the deceased [1]. Ritualistic cannibalism has also been thoroughly explored and documented throughout history in various tribes such as the Hurons and Iroquois of North America, the Ashanti of West Africa, and the Maori of New Zealand [1,16]. Understanding the cultural nuances of cannibalistic practices can provide crucial insight into the motivations and beliefs that drive such taboo behavior.

As elaborated in Cooper’s account of the Cree Witiko Psychosis in the Primitive Man, cannibalism was observed in the eastern Cree and other northern Canadian tribes and described as a typical psychosis known as “Witiko mala” [4]. To the Cree tribe and Algonquians, the practice of cannibalism was explained as a unique form of folk-lore psychosis. During times of famine, individuals suffering from mental illness and delusions were driven to crave human flesh and “transformed” into Witikos, beings with hearts of ice or who vomit ice [4]. Interestingly, these transformed individuals were subsequently killed, burned, and consumed, with the exception of their hearts, which were believed to be made of hard ice and destroyed [4]. Rather than interpreting the Wendigo phenomenon solely through a Eurocentric lens as a culture-specific psychiatric syndrome linked to extreme isolation and the stresses of a nomadic lifestyle, others argue that it can be viewed as an indigenous belief rooted in Algonkian culture, involving individuals in intimate contact with the intangible powers of the transcendental to achieve personal equilibrium within their social context [17].

These intricate cultural manifestations of cannibalism are further differentiated from psychopathologically-based acts of cannibalism, as evidenced in unique case studies involving auto-cannibalism. In one documented case, a 38-year-old man with a three-year history of paranoid schizophrenia, after a period of poor medical compliance and methamphetamine use, experienced intensified psychosis, including intense preoccupation with numbers and delusions of influence [18]. The patient proceeded to sever and consume his left and right small toes over the span of three weeks [18]. Despite the severity of the patient’s actions, he returned to work after the reinstatement of flupenthixol 20 mg IM monthly and olanzapine 5 mg per day, which stabilized his condition [18]. Another recent case study examined a rare instance of stress-induced auto-cannibalism following a week of self-destructive scratching as a means of self-soothing in a patient with a history of traumatic brain injury but no prior psychiatric history [19]. A similar report discussed a 43-year-old convict admitted for psychiatric evaluation due to self-inflicted removal and consumption of his abdominal tissue [20]. Unlike the other two case reports, this patient was hostile, irritable, and had severe delusions of persecution, which only subsided after a course of ten electroconvulsive therapy sessions [20]. While the ethical and moral dilemmas of cannibalism remain a topic of debate, it is important to distinguish between criminal cannibalism, self-cannibalism as a result of untreated psychiatric illness, and cannibalism as a survival or ritualistic mechanism rooted in cultural and religious motives, as seen in the context of Wendigo psychosis.

Contemporary relevance and cultural sensitivity

The phenomenon of Wendigo psychosis is a multifaceted and intricate occurrence that occurs within the yearly cycle of the Wendigo, involving the consumption of an individual by members of a dispersed, small extended family or camp [21]. Typically, the choice of victim is influenced by the perceived level of threat and the available amount of meat [21]. To better understand the complexities of Wendigo psychosis, it is important to consider the yearly cycle, the distribution of the Wendigo complex, and the cultural significance of the Wendigo myth within the societies that embrace it [21]. Further research should be conducted to explore the behavior of mentally balanced Northern Algonkian individuals concerning the Wendigo and the contemporary cultural implications of this phenomenon.

Addressing the topic of cannibalism with cultural sensitivity and respect is of paramount importance due to the intricate interplay between cultural practices, historical context, and the potential for misunderstanding or misrepresentation. It is essential to recognize that cannibalism takes on different meanings and manifestations in diverse cultural contexts. Approaching the subject with cultural sensitivity allows one to acknowledge the complexities of these practices without perpetuating harmful stereotypes or sensationalism. Without such sensitivity, there is a risk of reducing diverse cultural practices to a singular, distorted narrative that fails to capture the full depth of these traditions. Furthermore, careful consideration of the actors influencing cases of cannibalism is crucial for understanding why these practices emerged and the roles they played within specific cultures. It is also vital to note the lasting impact of historical trauma that misrepresentations of cannibalism in the media have had on Indigenous communities. Paradoxically, a study of real-life criminal cases involving cannibalistic acts found that the highest frequency of reported cannibalism cases in the 1960s was in the United States as compared to the rest of the modern world [22].

Furthermore, respectful engagement with the topic is vital to foster open dialogue and cross-cultural understanding. Cultural norms, beliefs, and rituals can be vastly different from one society to another, and interpreting them through a lens of empathy ensures that we do not impose our own judgments or preconceived notions onto these practices. Instead, it encourages us to appreciate the nuances and significance of cannibalistic practices within the frameworks in which they exist.

Psychopathology and human behavior

From the psychopathological perspective of cannibalism, many cases are concurrent with a psychiatric diagnosis, often some form of psychotic disorder. It is important to note, however, that mental disorders are not necessary or sufficient for instances of general violence or cannibalism [23]. Substance abuse, antisocial personality traits, and paraphilic behavior have often been underlying factors in cannibalistic acts, but not psychiatric disorders in general [23,24]. Additionally, in a study that compared biological, behavioral, social, and parental influences between cannibalistic serial killers and non-cannibalistic serial killers, cannibals were found to have come from lower socioeconomic backgrounds, have had more cases of abuse, and have more family members who were criminals [25]. This is indicative of cannibalism, as a subsequent act of serial killing is more closely associated with environmental upbringing and not purely a result of a primary psychiatric disorder. Furthermore, there have also been instances of both cannibalism and self-cannibalism (autosarcophagy) in the complete absence of psychiatric illness or substance abuse [26].

There have been a handful of cases involving acts of cannibalism or cannibalistic ideology in adults, but only one documented case in a child or adolescent [27]. Oldak et al. described the first known case of cannibalistic ideology in an adolescent 14-year-old female with no past psychiatric history who was referred to the emergency department for engaging in self-injurious behavior [27]. When evaluated, the patient was noted to also have severe depression, intrusive suicidal thoughts, long-standing perceptual disturbances of seeing the devil, and a self-reported two-year history of craving human flesh. The cannibalistic urges were stated to be mostly elicited by thoughts or interactions with those she did not like or felt were “against her” [27]. Interestingly, her symptoms seemed to have been precipitated by her current psychosocial stressors and history of severe sexual abuse or trauma [27]. While most reported cases of acts of cannibalism or urges to consume human flesh tend to be affiliated with a diagnosis of either psychosis or some form of paraphilia, this patient did not meet the criteria for either definitive diagnosis, and the psychiatric team suggested that it was more consistent with a diagnosis of complex post-traumatic stress disorder (PTSD) with some psychotic features likely stemming from her previous sexual abuse and trauma [27]. This is a departure from what has been seen in previous literature, where psychiatric patients who have engaged in cannibalistic behavior fall into one of two subgroups: severe schizophrenia/psychosis, where the patient engages in cannibalism in self-defense to perceived harm, and mixed personality disorder, where the individual has psychopathic and sadistic tendencies that lead to ego-syntonic cannibalistic behavior as a means of overcoming deep-rooted narcissistic frustrations [28].

Uniquely, the phenomenon of the Wendigo psychosis is not perpetuated by the cannibalistic urges seen in psychotic individuals of all societies, but instead, the peculiarities of this individual deviant behavior are a result of cultural and social factors as well as of individual psychodynamics [6]. The Northern Algonkian people who engage in eating human flesh are generally pursuing one of three outcomes: preserving a lost relationship, solving ambivalent feelings, or acquiring a property like vitality or courage [6]. Pursuit of one of these outcomes, often in combination with other outcomes such as nourishment for survival, seems to be involved in various disorders, such as windigo disorder, depression, schizophrenia, and ritualized cannibalism [6]. Furthermore, Brown hypothesized that the traditional "cure" of Wendigo psychosis for the Northern Alognkian people involved the ingestion of fatty meat, indicating that they felt that through sufficient nutrients, in this case, fats, the Wendigo psychosis that an individual was undergoing could be cured [29]. Due to the complexities of Wendigo psychosis and the intersection between its associated cultural and extra-cultural dimensions, there is a need for interdisciplinary involvement from numerous fields, including anthropology, forensic psychiatry, biology, and nutrition, to better understand how this cultural syndrome relates to psychiatric illness.

More insight and discussion on reported forensic cases of cannibalism should be portrayed in the media to elucidate the numerous nuances associated with this rare taboo act. Furthermore, forensic psychiatrists should seek to update the DSM-V to include the unique characteristics, nature, and psychological drive associated with cannibalistic acts, given that there is a considerable lack of information on how these individuals think [28]. One comprehensive review of cannibalism cases in the United States showed that there are consistent similarities among the individuals who have engaged in cannibalistic acts [28]. Many of these offenders had multiple victims who fit a certain criterion (i.e., white, middle-aged females) and only consumed certain body parts in a sexualized manner [28].

Other studies have further investigated the psychological motivations of those who engage in cannibalism and perpetrators of bite marks. The attack style, mode of death, and body part consumed all provide components to piecing together the inherent psychological needs that the offender is attempting to satisfy [30]. There seems to be a consistent underlying theme of desire for power, potency, and an “omniscient capacity for absorbing another’s life essence” that is at the root of psychopathological cannibalism [30]. Additionally, those in the field of forensic psychiatry must aim to separate the different types of serial killers and whether they engage in some form of cannibalistic act. One pilot study demonstrated that serial killers who engaged in both necrophilia and cannibalism were the most psychiatrically disturbed and deviant in their actions of all serial killers, but those who engaged in cannibalism alone were still far more disturbed and deviant than serial killers who did not engage in either behavior [31]. This suggests that psychiatric treatment for these individuals should be tailored based on the extent of their psychiatric disturbance and deviancy and that there should be further exploration into the unique nuances of these criminal offenders.

Paraphilia vs. Psychosis as the Underpinning of Cannibalistic Psychopathology

Cannibalism is often associated with some sort of paraphilia, particularly vorarephilia. Vorarephilia is a rare presenting paraphilia that involves an erotic desire to consume another individual’s flesh or, in turn, have one’s flesh consumed by another human or animal [32]. The only known reported psychiatric case of explicit vorarephilia was that of a man who presented to his local mental health clinic in Toronto, Canada, with concerns over his “sexual addiction” [32]. Upon psychiatric evaluation, in addition to his reported two primary interests, analingus and partialism (foot fetish), the patient had intense sexual masochistic fantasies of wanting to be consumed by a large, dominating woman and then be defecated by her [32]. While he reported no desire or urge to consume anyone himself, he did express sexual arousal at the thought of being a body part (an anus) and extreme hyper-fixation on becoming the end bodily product of feces [32]. This case is unique in that while his clinical presentation included elements of sexual masochism and vorarephilia, he diverged from the expected criteria through his intense desire for his human body to be destroyed and digested into human feces [32]. This seems to fall on the other end of the known cannibalistic psyche, where many who engage in this behavior have an intense desire and fascination with wanting to consume human flesh to exert dominance and overcome suppressed frustrations [27,32]. Since cannibalism and Wendigo psychosis are not discussed in the DSM-V, we would suggest that future iterations of the DSM include a section detailing the unique nuances of cannibalism, despite its rarity, and how it relates to certain elements of both paraphilic and psychotic disorders.

Psychoanalytic Perspective

Furthermore, cannibalistic fantasies can be attributed to some psychoanalytical mechanisms. Freud considered melancholia a severe form of depression where one recognizes an external loss and metaphorically consumes it to form an internalized grief [33]. This concept was further expanded by Julia Kristeva, who conceptualized cannibalistic fantasies into a melancholic paradigm where there is a denial of loss and the individual copes by destroying the source of their loss and consuming it to regain a sense of control [33]. Furthermore, it has been argued that many individuals who have engaged in cannibalistic behaviors are hyper-fixated on the oral stage of development due to unmet needs during childhood [34]. Given that the mouth is important in early development for nutritional functions and is the region where the beginning of psychosexual and ego development occurs [31], one can fathom how this psychological immobility from the oral stage of development could lead to cannibalistic thoughts and urges. These psychoanalytic perspectives can also be extended to cases of self-cannibalism, where an individual consumes their own hair, skin, or bodily fluids that they attribute meaning to, to disconnect destructively and then reconnect through consumption.

This association with the psychoanalytic cannibalistic connection can be explored further through less taboo psychiatric disorders such as trichophagia. Additionally, a practice that is becoming more common, especially in the United States, is eating postpartum placentas in some form due to their reported medicinal benefits [1]. It could be argued from a Freudian perspective that this action is a means for a mother to reconnect with the internalized sense of motherhood lost upon giving birth and that eating her placenta re-establishes the bond shared with her child. The same could be applied to what has been termed “medical cannibalism” regarding consumption of human breast milk and fecal transplants [35]. Breastmilk has been used for some therapeutic purposes, for the healthy survival of low-weight babies, and for medical research, while fecal transplants are often used for the restoration of the gut microbiome and as a possible treatment of ulcerative colitis [35]. There should be further exploration into the medicalization of human fluid consumption and the psychological aspects that might be at play in the societal acceptance of these profitable markets.

Popular culture

The media often sensationalizes cannibalistic crimes, resulting in heightened public interest in such cases [36,37]. However, sensationalism can perpetuate misconceptions about the motivations and mental states of those involved [38]. Studying the media's portrayal of cannibalism and its impact on public perceptions is essential to fostering a balanced understanding of these criminal acts.

Prior depictions of cannibalism in popular media have used the old racist depiction of cannibalism where the dark-skinned cannibal consumes the innocent Westerner [39]. Within the last few years, however, western media has capitalized on cannibalism by exploring, and often exaggerating, common horror tropes, but also, albeit less commonly, by romanticizing Hollywood depictions of those who have engaged in cannibalistic acts. The film "The Silence of the Lambs" portrays the horrifying and psychopathic desires associated with cannibalism [39]. Multiple depictions of Jeffrey Dahmer in movies and on television have portrayed him as a handsome yet deranged man and have sensationalized and fetishized his cannibalistic acts without taking a deeper dive into his motivations, upbringing, or mental state [40]. Bones and All, a 2015 novel by Camille DeAngelis, instead chooses to tell the story of two cannibal lovers where one ends up eating the other as the “ultimate depiction of love” [40].

Other depictions tend to look at cannibalism from a more scientific and naturalistic perspective. In his book, Cannibalism: A Perfectly Natural History, Bill Shutts discusses the biological functions of cannibalism as a behavior, from foraging to reproductive and parental necessities and environmental stresses [41]. This is further explained by a risk-benefit phenomenon where he claims that if any animal, including humans, is stressed far enough, they will eat their own kind if the benefit of survival outweighs the risk of catching a disease [41].

## Conclusions

The findings of our paper elucidate the intricate subject of cannibalism, spanning across diverse academic fields, including psychiatry, anthropology, psychology, and sociology. It primarily examines cannibalism through the prism of Wendigo psychosis, cultural interpretations, psychological factors, historical backgrounds, and contemporary implications. Wendigo psychosis, deeply embedded in the traditions of Algonquian-speaking tribes, serves as a cultural symbol that cautions against losing one's humanity during times of extreme hardship. This psychosis, marked by paranoia, anxiety, hallucinations, and cannibalistic impulses, often emerges from a blend of cultural narratives and psychological vulnerabilities. Historical records reveal that instances of Wendigo psychosis and cannibalism were more common during periods of severe famine among these tribes. Cannibalism itself takes various forms, from ritualistic to survival-driven, across different cultures, underscoring the need for cultural sensitivity and respect. Approaching this topic with empathy fosters open dialogue and promotes cross-cultural understanding. Furthermore, it is essential to recognize that cannibalism is not solely linked to mental illness but can result from factors like substance abuse, environmental upbringing, and survival instincts in response to trauma.

Understanding the psychological aspects of cannibalism, encompassing mechanisms such as melancholia and oral fixation, can provide insights into the motivations behind such behavior. The paper also highlights the impact of media portrayals, which can distort public perceptions of cannibalism. In sum, this research underscores the need for a multidisciplinary approach to comprehend cannibalism's cultural, psychological, and historical dimensions. Sensitivity and respect are paramount in discussing this complex subject, given the diverse motivations and manifestations of cannibalistic behavior, whether within the context of Wendigo psychosis or other cultural practices.
